# Risk prediction model for parastomal hernia after colorectal cancer surgery: systematic review and meta-analysis

**DOI:** 10.1186/s12893-026-03782-7

**Published:** 2026-04-27

**Authors:** Siyu Liu, Lintong Wang, Jin Zheng, Min Ju

**Affiliations:** 1 Department of Nursing, the First Affiliated Hospital of China Medical University, Shenyang, Liaoning Province People’s Republic of China; 2 Department of Nursing, the Fourth Affiliated Hospital of China Medical University, Shenyang, Liaoning Province People’s Republic of China; 3 Department of Urology, the First Affiliated Hospital of China Medical University, Shenyang, Liaoning Province People’s Republic of China

**Keywords:** Parastomal Hernia, Risk prediction model, Systematic review

## Abstract

**Objective:**

To systematically assess risk prediction models for Parastomal Hernia after colorectal cancer surgery.

**Methods:**

We searched databases until October 2025 for studies developing or validating Parastomal Hernia prediction models. Study quality was evaluated using the PROBAST tool. Model performance measures were pooled via meta-analysis, while predictors were summarized qualitatively using narrative synthesis.

**Results:**

Seven retrospective studies were included. Four studies contributed to the internal validation pool, yielding a high pooled discrimination (AUC = 0.910). Two studies contributed to the external validation pool, showing good performance (AUC = 0.854). Key predictors consistently identified included stoma diameter, age, sex, BMI, and albumin. All studies were rated at high risk of bias due to retrospective design and methodological limitations.

**Conclusion:**

Current prediction models show promising discriminatory performance but are at high risk of bias and have undergone limited external validation. Their clinical applicability remains uncertain. Future research requires rigorous prospective development and broader validation.

**Systematic review registration:**

PROSPERO CRD420251168201.

**Supplementary Information:**

The online version contains supplementary material available at 10.1186/s12893-026-03782-7.

## Introduction

According to 2022 global cancer statistics, colorectal cancer is the third most common malignant tumor worldwide and the second leading cause of cancer-related mortality [1]. Currently, surgical resection remains the primary curative treatment, with Parastomal Hernia (PSH) being one of the most frequent long-term complications after stoma formation [2]. PSH is defined as an abnormal protrusion of abdominal contents through a fascial defect in the abdominal wall surrounding the stoma [3]. Its reported incidence varies depending on the duration of follow-up and the diagnostic criteria used for PSH. The incidence of PSH is lower following ileostomy compared to urostomy and colostomy [4]. PSH not only causes local bulging, pain, stoma leakage, anxiety, and depression, significantly impairing patients’ quality of life [5, 6], but may also lead to serious complications such as intestinal obstruction, incarceration, and even strangulation [7, 8]. Consequently, patients often need to change their ostomy bags frequently, increasing their financial burden, and some may even require surgical intervention [9, 10]. Furthermore, even after undergoing PSH repair, there remains a high risk of recurrence and reoperation [11], imposing a substantial healthcare burden on both patients and society.

Given the high incidence and significant clinical impact of PSH, accurately identifying high-risk patients and implementing targeted preventive strategies is crucial. In recent years, researchers have developed various risk prediction models based on patient baseline characteristics, perioperative data, and surgical details, aiming to quantitatively assess the individual risk of developing PSH. If effectively validated, such models could assist clinicians in achieving precise preoperative risk stratification. This would enable more aggressive preventive measures for high-risk patients (e.g., prophylactic mesh reinforcement [12], optimized stoma site selection [13]), while avoiding unnecessary interventions for low-risk individuals. However, research in this field still faces numerous challenges. Existing prediction models incorporate different predictor variables and assign varying weights to them, resulting in inconsistent model performance. Currently, there is a lack of comprehensive studies that systematically evaluate, compare, and integrate existing risk prediction models for PSH after colorectal cancer surgery.

Therefore, this study aims to systematically identify and evaluate published risk prediction models for PSH following colorectal cancer surgery. By assessing the methodological quality and predictive performance of existing models, we seek to clarify the current evidence landscape and identify robust predictors. Ultimately, this review aims to provide a consolidated evidence base to inform future research and, more importantly, to guide clinical decision-making. The findings are intended to assist clinicians in key perioperative decisions, such as personalized preoperative patient counseling regarding PSH risk, selective use of prophylactic mesh reinforcement in high-risk individuals, and optimization of stoma site selection techniques. A clear understanding of reliable predictive tools is a crucial step toward reducing the incidence of this common complication.

## Methods

### Search strategy

A comprehensive literature search was undertaken in nine electronic databases, including CNKI, Wanfang, VIP, CBM, PubMed, Web of Science, Cochrane Library, EBSCO, and Embase, covering the period from database inception to October 1, 2025. The specific search strategies are elaborated in Supplementary Material 1. To further ensure literature saturation, we also performed a manual snowball search by reviewing the reference lists of all included studies. This systematic review of prediction models was conducted and reported in accordance with the CHARMS guidelines. The conduct of this review was informed by the PICOTS framework (Supplementary Material 2) and the CHARMS checklist for data extraction (Supplementary Material 3). This study was registered with the International Prospective Register of Systematic Reviews (PROSPERO) under registration number CRD420251168201.

### Inclusion and exclusion criteria

Inclusion Criteria were as follows: (a) study population of adults with an ileostomy or colostomy created after colorectal cancer surgery; (b) development and/or validation of a prediction model for PSH risk; and (c) PSH included as an outcome. Exclusion Criteria included: (a) studies only reporting risk factors without model construction; (b) models with fewer than two predictors; (c) unavailability of the full text; (d) theses or dissertations; and (e) studies published in languages other than Chinese or English.

### Study screening and data collection

After deduplication of the search results was undertaken with EndNote X22, study selection and data extraction were carried out independently by two investigators using a pre‑specified, standardized data extraction form based on the CHARMS checklist [14]. The same two investigators independently performed the quality assessment using the PROBAST tool. A pre‑specified data extraction form was utilized to collect information, including sources of data, characteristics of the study participants, outcomes, predictors, sample size, statistical methods, model predictive performance, as well as model evaluation and presentation. All extractions and quality assessments were rigorously cross‑checked between the two reviewers. In case of disagreements, adjudication by a third reviewer was sought to achieve consensus.

### Risk of bias and reporting transparency assessment

We evaluated the risk of bias and applicability of the included studies using the PROBAST, which assesses the quality and potential risk of bias in the selected studies [15]. To assess the completeness of reporting, we referenced the TRIPOD statement as a reporting guideline framework [16]. This tool evaluates the risk of bias across four domains: participants, predictors, outcomes, and statistical analysis, while also assessing the applicability of the first three domains. Responses for each domain were categorized as low, high, or unclear. If at least one domain was rated as having a high risk of bias or high concern regarding applicability, the overall assessment was considered high risk or high concern for applicability. If at least one domain was rated as unclear, with no domains rated as high risk or high concern for applicability, the overall assessment was deemed unclear. The evaluations were conducted independently by two researchers, and any discrepancies were resolved through discussion with a third researcher.

### Data analysis

We employed descriptive analysis to synthesize the basic characteristics, development methods, validation strategies, and predictors of the included prediction models. To quantitatively synthesize the predictive performance of the models, we conducted a meta-analysis using MedCalc (version 23.3.4).

For the synthesis of model discrimination metrics, our objective was to comprehensively evaluate model performance across different validation tiers. Consequently, for studies reporting both internal and external validation results, discrimination metrics from both validation cohorts were included in the pooled analysis; for studies reporting only a single validation cohort, data from that cohort were extracted. The extracted metrics included the area under the receiver operating characteristic curve (AUC) or the time-dependent concordance index (C-index), along with their 95% confidence intervals (CI). Although derived from different modeling frameworks, both metrics share the same statistical interpretation in measuring a model’s ability to discriminate between outcomes and were therefore treated as comparable effect measures for pooling. If a study developed or compared multiple models, data from the model demonstrating the best discrimination on the validation set were selected for analysis. For studies reporting C-index values at multiple time points, the value closest to the reported median follow-up time was chosen. The standard error (SE) for each effect measure was derived from its 95% confidence interval using the formula: SE = (CI upper-CI lower) / (2 × 1.96). Subsequently, a random-effects model based on the generic inverse-variance method was applied to pool the effect measures. Heterogeneity across studies was assessed using the I² statistic, interpreted as follows: I² ≤ 25% indicating low heterogeneity, 25% < I² ≤ 50% indicating moderate heterogeneity, and I² > 50% indicating high heterogeneity. The discriminatory ability of the models was interpreted based on the pooled AUC value: 0.7–0.9 indicates moderate predictive ability, and > 0.9 indicates high predictive accuracy.

Due to heterogeneity in how predictors were handled across the included studies, a quantitative pooling of their effect estimates was deemed methodologically unsound. Therefore, we forwent a traditional predictor-focused meta-analysis and instead conducted a narrative synthesis for exploratory purposes to identify potential candidate variables and assess the consistency of their reported associations.

## Results

### Selection of studies

**Fig. 1:**
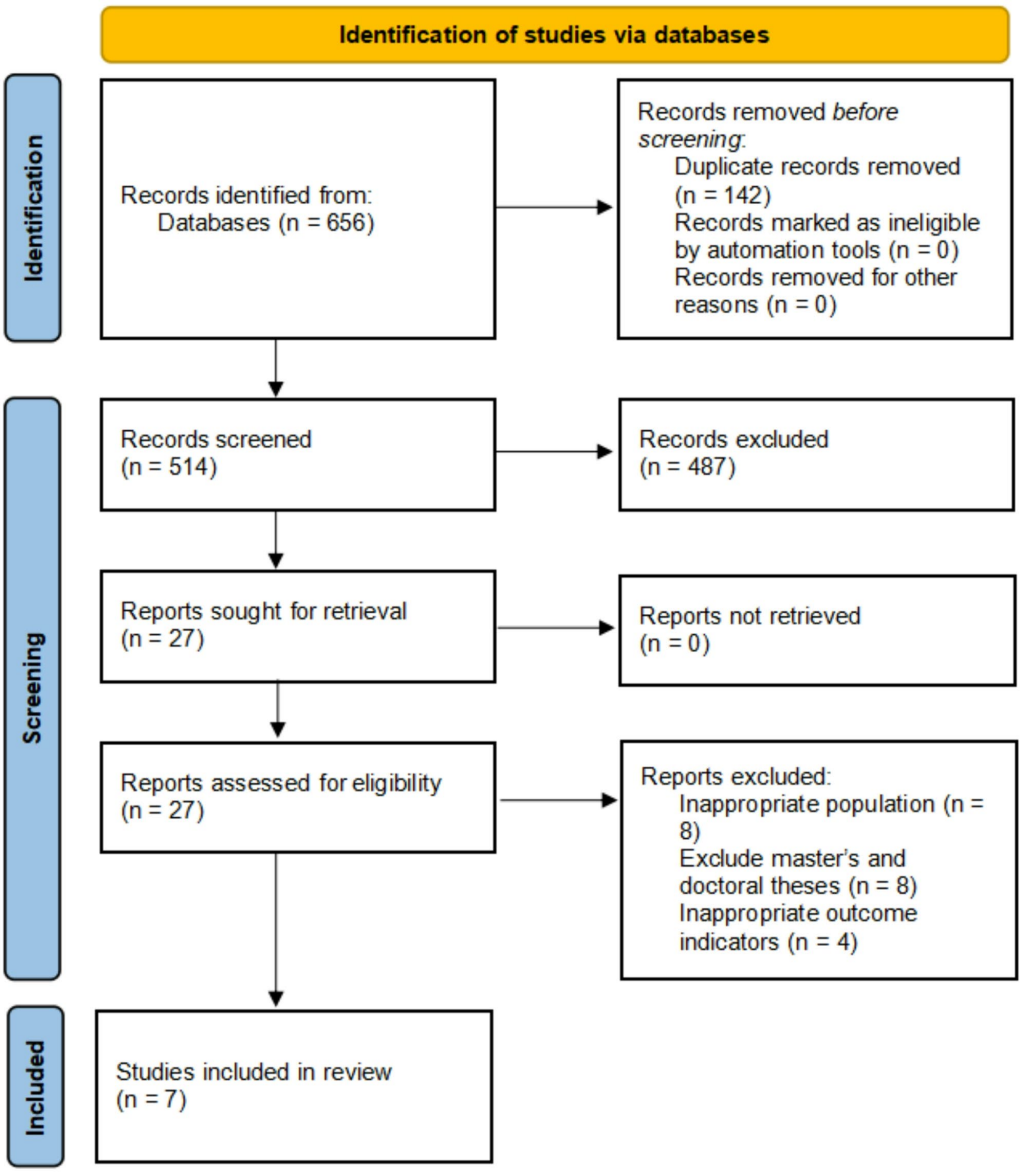
Flow diagram of the study selection process

Figure 1 illustrates the study selection process following the PRISMA 2020 guidelines. A total of 656 records were identified through database searches. After removing 142 duplicates, 514 records underwent title and abstract screening, with 487 excluded as irrelevant. The full texts of the remaining 27 articles were assessed for eligibility. At this stage, 8 studies were excluded due to inappropriate population, 8 were excluded master’s and doctoral theses, and 4 were excluded due to inappropriate outcome indicators. Finally, 7 studies met the inclusion criteria and were included in the systematic review and analysis.

### Study characteristics

Seven retrospective studies published between 2024 and 2025 [17–23], all conducted in China, investigated colorectal cancer patients who underwent various types of colostomies, such as end colostomies [18, 21], permanent sigmoid colostomies [17, 19, 20, 22], or prophylactic colostomies [23]. The sample sizes ranged from 131 to 836 patients, with follow-up periods varying from 1 year to 108 months. The incidence of PSH ranged from 5.3% to 32.8%. The lowest incidence was observed in patients with prophylactic colostomies [23], while the highest incidence was reported in those with end colostomies [18]. The baseline characteristics of the included studies are presented in Table 1.

**Table 1:** Basic characteristics of included studies (*n* = 7)

Author (year)	Country	Study Design	Participants	Sample size			Follow-up time	Incidence of parastomal hernia
				D	IV	EV		
Li et al. [18] (2024)	China	Retrospective	Patients with rectal cancer or sigmoid colon cancer undergoing end colostomy	92	39	/	24–80 months	32.8%
Liu^A^et al. [17] (2024)	China	Retrospective	Patients with a permanent sigmoid colostomy	/	291	63	2 years	20.96%/31.75%
Shi et al. [19] (2025)	China	Retrospective	Patients with colorectal cancer and a permanent colostomy	316	/	/	36 months	31.01%
Dai et al. [20] (2024)	China	Retrospective	Patients with colorectal cancer and a permanent colostomy	/	495	/	1 year	29.1%
Li et al. [22] (2025)	China	Retrospective	Patients with rectal cancer and a permanent colostomy	836	/	/	48–108 months	24.8%
Liu^B^et al. [21] (2024)	China	Retrospective	Patients with rectal adenocarcinoma undergoing permanent end colostomy	/	302	67	2 years	20.9%/31.3%
Yang et al. [23] (2025)	China	Retrospective	Patients with rectal cancer receiving prophylactic colostomy	405	174	/	At least 18 months	5.3%

*D* Development Set, *IV* Internal validation Set, *EV* External validation Set

Superscript letters A and B distinguish two distinct studies by the same first author (Liu) published in the same year (2024)

### Predictive model construction

Among the included predictive modeling studies (Table 2), the sample sizes ranged from 131 to 836, with the number of positive cases varying between 31 and 207. The number of candidate predictor variables spanned from 11 to 48. Variable selection methods were diverse, with most studies employing univariate analysis followed by multivariate analysis [17, 19, 21, 22], while some applied LASSO regression [20] or the Boruta algorithm [23] for feature selection. Continuous variables were primarily handled either by direct inclusion [17, 18, 21, 23] or transformation into categorical variables [19, 20, 22]. Regarding missing data, only two studies explicitly described their handling approaches, utilizing multiple imputation [20] and complete case analysis [23], respectively. Model development methods were relatively diverse; in addition to conventional approaches such as logistic regression (LR) [17, 20, 21, 23] and cox proportional-hazards model (Cox) [19, 22], several studies also extensively employed various machine learning algorithms, including support vector machines (SVM) [18, 23], decision trees (DT) [18], random forests (RF) [18, 20, 23], k-nearest neighbors (KNN) [20, 23], as well as ensemble models such as light gradient boosting machine (LightGBM) [20] and extreme gradient boosting (XGBoost) [20, 23].

**Table 2:** Information on prediction models in included studies (*n* = 7)

Author (year)	Candidate variable			Total sample size						Missing data		Modeling Methods
	Potential predictor	Variable selection	Continuous variable handling	D		IV		EV		Number of cases	Processing Method	
				Number of cases	Positive Cases	Number of cases	Positive Cases	Number of cases	Positive Cases			
Li et al. [18] (2024)	17	Univariate analysis	Continuous variable	92	31	39	12	/	/	/	/	SVM、DT、RF
Liu^A^et al. [17] (2024)	17	Univariate analysis Multivariate analysis	Continuous variable	/	/	291	61	63	20	/	/	LR
Shi et al. [19] (2025)	22	Univariate analysis Multivariate analysis	Categorical variables	316	98	/	/	/	/	/	/	Cox
Dai et al. [20] (2024)	48	LASSO regression with 10-fold cross-validation	Categorical variables	/	/	495	144	/	/	/	Multiple imputation method	LR、SVC、KNN、RF、LightGBM、XGBoost
Li et al. [22] (2025)	15	Univariate analysis Multivariate analysis	Categorical variables	836	207	/	/	/	/	/	/	Cox
Liu^B^et al. [21] (2024)	11	Univariate analysis Multivariate analysis	Continuous variable	/	/	302	63	67	21	/	/	LR
Yang et al. [23] (2025)	20	Boruta algorithm	Continuous variable	405	21	174	10	/	/	/	Excluded patients with incomplete or insufficient follow-up data	RF、LR、KNN、 XGBoost、SVM

*D* Development Set, *IV* Internal validation Set, *EV* External validation Set, *SVM* Support Vector Machine, *DT* Decision Tree, *RF* Random Forest, *LR* Logistic Regression, *Cox* Cox Proportional Hazards Model, *SVC* Support Vector Classification, *KNN* K-Nearest Neighbors, *LightGBM* Light Gradient Boosting Machine, *XGBoost* Extreme Gradient Boosting

Superscript letters A and B distinguish two distinct studies by the same first author (Liu) published in the same year (2024)

### Predictive model performance and presentation

**Fig. 2:**
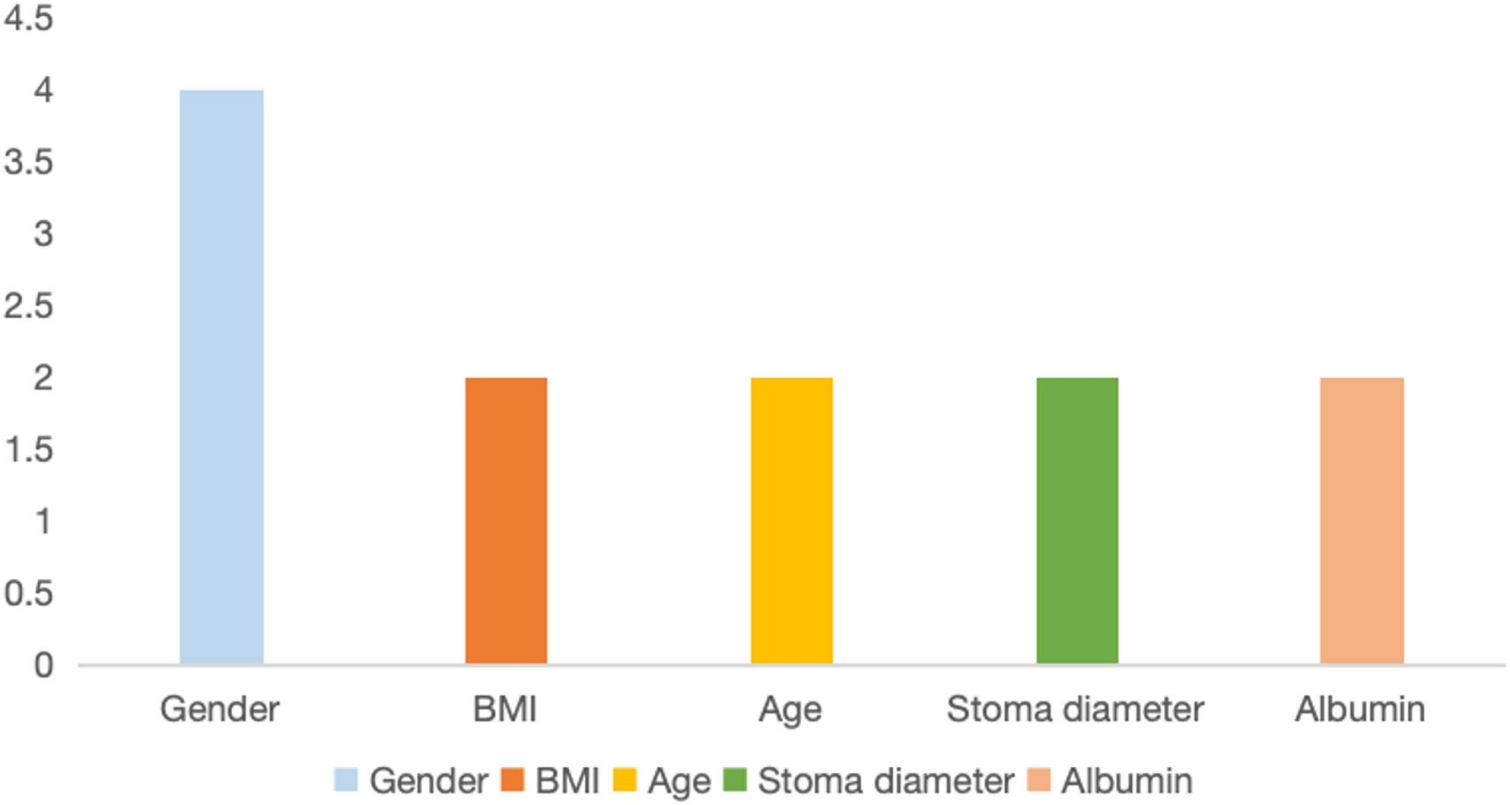
Predictors with higher frequency

This study included seven risk prediction models (Table 3). In terms of model performance, most studies demonstrated good discriminative ability, with AUC or C-index generally above 0.80. Notably, the models developed by Liu et al. [17] and Yang et al. [23] showed particularly outstanding performance, achieving C-index/AUC values of 0.941 and 0.909, respectively. Regarding validation strategies, some studies [17, 21] performed both internal and external validation, and their models maintained good predictive performance in external validation cohorts. For calibration assessment, Liu et al. [17, 21]used the Hosmer-Lemeshow test, while Liu et al. [21]and Yang et al. [23]employed calibration curves. The predictors incorporated in the models mainly included clinical indicators such as Body Mass Index (BMI) [17–23], age [17, 19, 21, 22], gender [17, 19, 21, 22], albumin [17, 20], and stoma diameter [17, 20–22], as well as radiomics features [18] (Fig. 2). The final models were mostly presented as nomograms [17, 19–22], with some studies also incorporating SHAP plots [20, 23] or online prediction platforms [23] to enhance model interpretability and clinical applicability.

**Table 3:** Performance and validation of risk prediction models (*n* = 7)

Author (year)	Model performance AUC/C-index			Calibration method	Model validation	Predictors	Model presentation
	D	IV	EV				
Li et al. [18] (2024)	SVM: AUC = 0.820 (95% CI: 0.662–0.978)	SVM: AUC = 0.804 (95% CI: 0.640–0.967)	/	/	IV	Six imaging features (2 shape features [sphericity, surface area] and 4 texture features [gray-level variance, gray-level non-uniformity, area percentage, coarseness]) and 2 clinical indicators (BMI and total serum protein)	/
Liu^A^et al. [17] (2024)	/	C-index 0.941 LR: AUC = 0.941 (95% CI: 0.913–0.970)	C-index 0.887 LR: AUC = 0.887 (95% CI: 0.800−0.975)	H-L	IV; EV	Age, sex, BMI, albumin, stoma diameter, subcutaneous fat index, rectus abdominis index	Nomogram
Shi et al. [19] (2025)	COX: AUC = 1 year: 0.744, 2 years: 0.834, 3 years: 0.932	/	/	/	D	Age, sex, BMI, diabetes mellitus, major postoperative complications	Nomogram
Dai et al. [20] (2024)	/	RF: AUC = 0.888 (95% CI: 0.881–0.935)	/	/	IV	BMI, Operation duration, history and status of COPD, prealbumin, TNM staging, stoma site, thickness of rectus abdominis muscle, c-reactive protein, ASA classification, stoma diameter	Nomogram, SHAP plots
Li et al. [22] (2025)	COX: AUC = 1 year: 0.650, 2 years: 0.740, 3 years: 0.760, 4 years: 0.790, 5 years: 0.830	/	/	/	D	Age, sex, BMI, transperitoneal stoma route	Nomogram
Liu^B^et al. [21] (2024)	/	C-index: 0.909 LR: AUC = 0.909 (95% CI: 0.874–0.943)	C-index: 0.801 LR: AUC = 0.801 (95% CI: 0.683–0.920)	CC; H-L	IV; EV	Age, sex, BMI, stoma diameter, albumin	Nomogram
Yang et al. [23] (2025)	RF: AUC = 0.909 (95% CI: 0.844–0.974)	RF: AUC = 0.900 (95% CI Not Reported)	/	CC	IV	Tumor distance from anal verge, BMI, hypertension, albumin	Online predictive platform, SHAP plots

*D* Development Set, *IV* Internal validation Set, *EV* External validation Set, *AUC* Area Under Curve, *CI* Confidence Interval, *SVM* Support Vector Machine, *LR* Logistic Regression, *Cox* Cox Proportional-Hazards Model, *RF* Random Forest, *H-L* Hosmer-Lemeshow, *CC* Calibration Curve, *BMI* Body Mass Index, *COPD* Chronic Obstructive Pulmonary Disease, *ASA* American Society of Anesthesiologists Physical Status Classification, *SHAP* SHapley Additive exPlanations

Superscript letters A and B distinguish two distinct studies by the same first author (Liu) published in the same year (2024)

### Quality assessment and risk of bias in the included studies

**Fig. 3:**
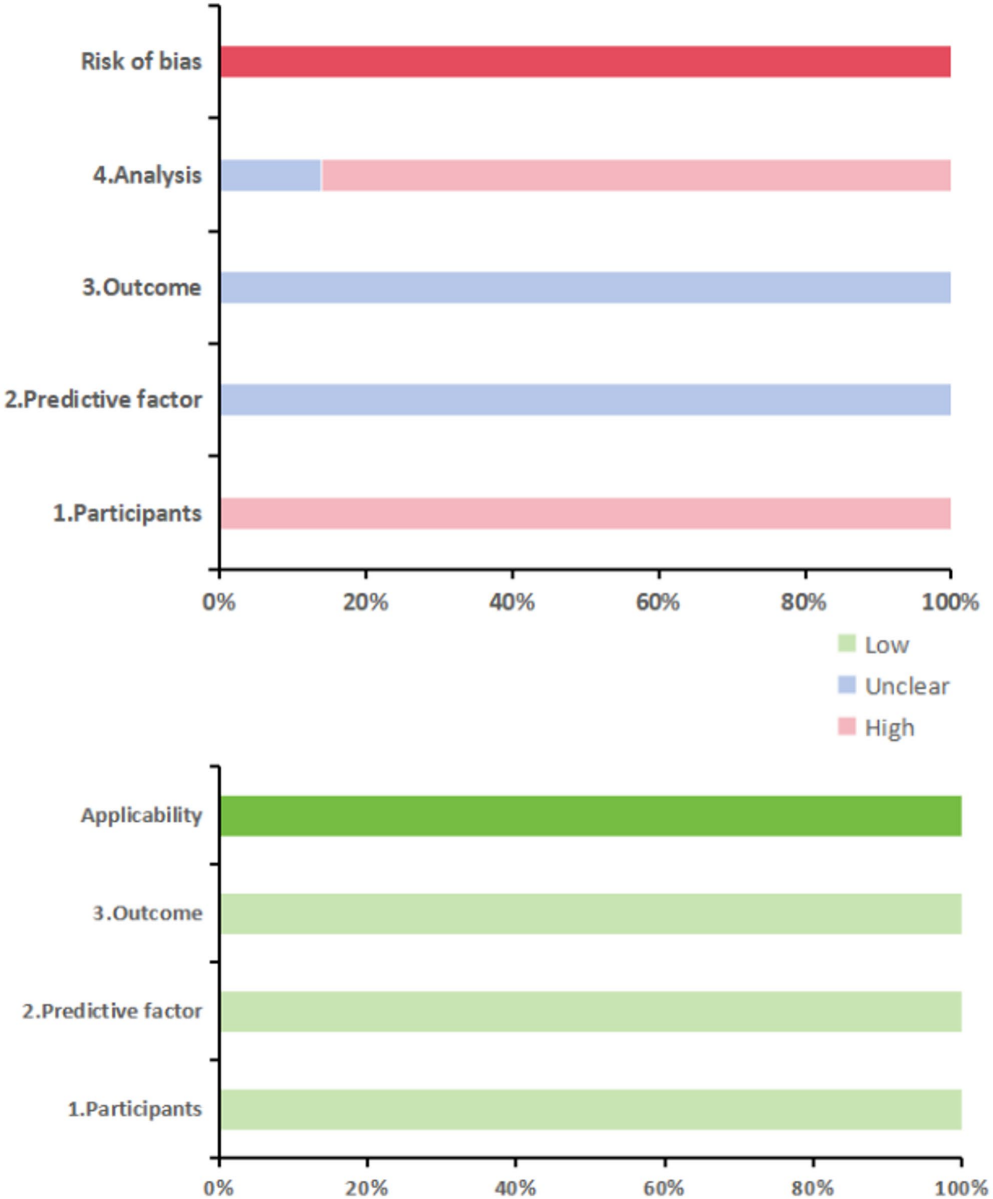
Summary of risk of bias and applicability evaluation (PROBAST)

All included studies were rated as having an overall “high” risk of bias, whereas concerns regarding applicability were rated as “low” (Table 4; Fig. 3 and Supplementary Material 4). The assessment of the risk of bias strictly followed the four domains of PROBAST. In the domain of participants, all studies were rated as high risk due to their retrospective cohort design. In the domain of predictors, all studies were rated as having an unclear risk because it was not explicitly reported whether the assessment of predictors was conducted under blinding. In the outcome domain, all studies were also rated as unclear risk as they did not report whether the outcome determination process involved blinded assessment. In the analysis domain, except for one study that was rated as “unclear” risk due to insufficient explanation of data complexity [20], all remaining studies were rated as “high” risk. These high-risk ratings primarily stemmed from the following methodological deficiencies: five studies had an inadequate sample size [17–19, 21, 23]; one study converted continuous variables into categorical variables [22]; six studies reported insufficient methods for handling missing data [17–19, 21–23]; five studies relied solely on univariate analysis to select predictors [17–19, 21, 22]; all studies failed to adequately consider or report issues related to data complexity; two studies did not perform internal validation [19, 22]; and one study did not report information on the consistency between regression coefficients and the results [18]. All studies demonstrated high alignment with the research question of this systematic review in terms of the definitions of participants, predictors, and outcomes. Consequently, the overall concerns regarding applicability were low.

**Table 4:** Assessment of included studies’ Bias risk and applicability

Author(year)	Risk of bias				Applicability			Overall	
	Participants	Predictive factor	Outcome	Analysis	Participants	Predictive factor	Outcome	Risk of bias	Applicability
Li et al. [18] (2024)	**-**	?	?	-	+	+	+	-	+
Liu^A^et al. [17] (2024)	**-**	?	?	-	+	+	+	-	+
Shi et al. [19] (2025)	**-**	?	?	-	+	+	+	-	+
Dai et al. [20] (2024)	**-**	?	?	?	+	+	+	-	+
Li et al. [22] (2025)	**-**	?	?	-	+	+	+	-	+
Liu^B^et al. [21] (2024)	**-**	?	?	-	+	+	+	-	+
Yang et al. [23] (2025)	**-**	?	?	-	+	+	+	-	+

*ROB* Risk Of Bias

+ Low ROB/Low concern regarding applicability

- High ROB/High concern regarding applicability

? Unclear ROB/Unclear concern regarding applicability

Superscript letters A and B distinguish twodistinct studies by the same irst author (Liu) published in the same year (2024)

### Model performance validation and narrative synthesis of predictors

The model performance validation results (Supplementary Material 5) showed that the pooled AUC for internal validation [17, 18, 20, 23]was 0.909 (95% CI: 0.876–0.942), indicating high discriminative ability. Heterogeneity testing suggested moderate heterogeneity (I²= 65.59%, *P* < 0.001). For external validation [17, 21], the pooled AUC was 0.854 (95% CI: 0.772–0.936), reflecting moderate discriminative ability with low heterogeneity (I²= 23.62%, *P* < 0.001). A comprehensive analysis was conducted on the five most frequently reported predictors (age, sex, BMI, stoma diameter, albumin) in the existing literature (Table 5). The results demonstrated that higher BMI [16–22] and larger stoma diameter [16, 19–21] were consistently identified as significant risk factors for PSH in all models that included these variables. Furthermore, advanced age [16, 18, 20, 21] and female [16, 18, 20, 21] were also associated with an increased risk of PSH in most studies, while higher preoperative serum albumin levels were related to a reduced risk [16, 19]. However, notable heterogeneity was observed in the effect sizes and statistical significance of these associations.

**Table 5:** Variable handling and effect size of frequently reported predictors

Predictors	Author (year)	Variable handling	Effect size
BMI	Li et al. [18] (2024)	Cont.	*P* = 0.044
	Liu^A^et al. [17] (2024)	Cont.	OR = 1.553
	Shi et al. [19] (2025)	Cont.	HR = 1.933
	Dai et al. [20] (2024)	Cat.	/
	Li et al. [22] (2025)	Bin.	HR = 2.100
	Liu^B^et al. [19] (2024)	Cont.	OR = 1.360
	Yang et al. [23] (2025)	Cont.	/
Age	Liu^A^et al. [17] (2024)	Cont.	OR = 1.063
	Shi et al. [19] (2025)	Cont.	HR = 2.411
	Li et al. [22] (2025)	Bin.	HR = 5.170
	Liu^B^et al. [21] (2024)	Cont.	OR = 1.047
Sex	Liu^A^et al. [17] (2024)	Bin.	OR = 3.927
	Shi et al. [19] (2025)	Bin.	HR = 2.542
	Li et al. [22] (2025)	Bin.	HR = 2.280
	Liu^B^et al. [21] (2024)	Bin.	OR = 6.566
Stoma diameter	Liu^A^et al. [17] (2024)	Cont.	OR = 4.426
	Dai et al. [20] (2024)	Bin.	/
	Liu^B^et al. [21] (2024)	Cont.	OR = 7.785
Albumin	Liu^A^et al. [17] (2024)	Cont.	OR = 0.876
	Liu^B^et al. [21] (2024)	Cont.	OR = 0.850

*Cont.* Continuous variable, *Bin.* Binary variable, *Cat.* Categorical variable, *OR* Odds Ratio, *HR* Hazard Ratio

Superscript letters A and B distinguish two distinct studies by the same first author (Liu) published in the same year (2024)

## Discussion

This study systematically reviewed and meta-analyzed recent advances in risk prediction models for PSH after colorectal surgery. The results indicate that although several models have demonstrated good predictive performance, their methodological quality and clinical applicability still face challenges.

### Model performance and clinical translation

In terms of discriminative ability, most models showed promising results. The pooled AUC for internal validation was 0.909, suggesting that existing models can potentially distinguish between high risk and low risk patients. The pooled AUC for external validation was 0.854, indicating a moderate degree of generalizability for some models. However, an overemphasis on discrimination is insufficient for clinical adoption. For a model to be useful in daily practice, it must inform specific decisions. For instance, a model with high discrimination could be used preoperatively to counsel a patient about their individual risk, helping them weigh the benefits of a stoma against the likelihood of a future hernia. More importantly, it could guide the selective use of prophylactic mesh reinforcement: a high-risk prediction might justify this more invasive intervention, while a low-risk prediction would spare the patient from potential mesh-related complications. Critical appraisal reveals a striking gap in the reporting and assessment of calibration and clinical utility. Only a minority of studies employed calibration curves or the Hosmer-Lemeshow test [16, 20, 22], and none conducted decision curve analysis to quantify the net benefit of using the model for clinical decision-making across different risk thresholds. The absence of these evaluations limits our understanding of whether these models provide accurate absolute risk estimates and whether acting on their predictions would improve patient outcomes.

### Synthesis for common predictors and limitations of existing models

Our narrative synthesis identified stoma diameter, BMI, age, sex, and albumin as the most frequently reported predictors. It must be emphasized that due to significant heterogeneity across studies in the definitions, measurement methods, coding, sets of adjusted covariates, and variable selection procedures for these predictors, a quantitative meta-analysis of their effect estimates was not conducted in this review, as such pooling could yield misleading interpretations. An enlarged stoma diameter directly increases the abdominal wall defect and exacerbates circumferential tension, thereby delaying fascial healing [24]. This finding underscores the critical importance of creating a properly sized stoma during surgery [25]. Aging is associated with reduced strength and elasticity of the abdominal wall muscles and fascial tissues, as well as diminished repair capacity [26]. Furthermore, females had an increased risk, which may be attributed to their generally higher percentage of subcutaneous fat and thinner abdominal wall muscles compared to men [27]. Elderly patients often present with multiple comorbidities (e.g., chronic cough, constipation, avoid strenuous activity and exercise), and the resulting increase in intra-abdominal pressure further exacerbates the mechanical load around the stoma [28, 29]. Albumin demonstrated a protective effect. This association may reflect the positive impact of nutritional status on tissue healing capacity [30], thereby supporting preoperative nutritional support as a potential preventive strategy [31].

However, the predictor frameworks of existing models exhibit significant limitations, as they fail to adequately incorporate key context-specific factors that are closely linked to the pathogenesis of PSH and particularly relevant to colorectal cancer. Regarding surgical factors, the specific operative approach, such as abdominoperineal resection versus low anterior resection, alters the biomechanical environment at the stoma site but is frequently overlooked. Similarly, the route of stoma construction, whether extraperitoneal or transperitoneal, is a well-established variable influencing hernia risk yet is seldom represented in these models. In terms of patient and treatment characteristics, neoadjuvant chemoradiotherapy, a standard treatment for rectal cancer, may potentially compromise parastomal tissue strength by inducing fibrosis and vascular injury, but this critical therapeutic dimension is also missing. Furthermore, assessments of body composition and sarcopenia, which more accurately reflect metabolic reserve and muscular health than body mass index alone, have not been utilized in current models. Most importantly, prophylactic mesh reinforcement, an effective preventive intervention, has neither been included as a predictor nor considered as an effect modifier, which undoubtedly limits the models’ predictive accuracy in clinical practice. In light of these limitations, future prediction models should prioritize the inclusion of these context-specific factors. Incorporating variables such as surgical approach, stoma route, neoadjuvant chemoradiotherapy, sarcopenia, and prophylactic mesh reinforcement would not only improve model accuracy but also enhance clinical relevance and applicability across diverse patient populations and treatment settings.

### Methodological limitations

However, despite the favorable predictive performance of the models, methodological limitations were prevalent. All included studies were rated as having a high risk of bias, with issues primarily concentrated in patient selection and statistical analysis. Retrospective design, insufficient sample size, misclassification of continuous variables, improper handling of missing data, and inadequate variable selection methods may all compromise the robustness and reliability of the models. Furthermore, most studies did not adequately assess or report the calibration performance of the models, with only a few employing calibration curves or the Hosmer–Lemeshow test, limiting a comprehensive understanding of their predictive accuracy. Notably, although most studies employed traditional modeling methods such as logistic regression or Cox regression, some attempted to incorporate machine learning algorithms (e.g., random forest, XGBoost, LightGBM), demonstrating excellent performance. Additionally, several studies enhanced the clinical usability and interpretability of the models through nomograms, SHAP plots, or online prediction platforms, reflecting a trend toward the clinical translation of predictive models.

### Clinical practice and future directions

Based on the comprehensive analysis of this review, due to prevalent methodological limitations and the lack of robust external validation, no single model can currently be recommended for direct use in routine clinical practice. Future research should focus on developing predictive tools with clear clinical translational value. This entails moving beyond a sole emphasis on discriminative ability and further integrating model outputs with actionable clinical interventions. For example, studies have shown that prophylactic mesh reinforcement can reduce the incidence of PSH in high-risk populations [32]. An ideal predictive model should accurately identify the subgroup of patients who would derive the greatest net benefit from such interventions, thereby enabling personalized prevention strategies. Furthermore, models could guide preoperative prehabilitation (e.g., optimizing nutritional support for patients with hypoalbuminemia) or assist in intraoperative decision-making (e.g., selection of stoma siting techniques). The influence of surgical factors must also be considered. Future models should account for variations in surgical technique, such as the specific operation performed and the route of stoma creation. Additionally, the role of surgeon experience, including the learning curve and standardization of stoma construction, is a critical variable that can significantly impact outcomes and should be explored as a potential predictor or a factor in model calibration. Therefore, prospective model development and rigorous external validation are essential foundations. Future models should explicitly incorporate the colorectal cancer-specific factors discussed above to enable effective risk-stratified precision management and improve patient prognosis.

## Limitations

This study has several limitations. First, all included studies were retrospective in design and based solely on Chinese populations, which may limit the applicability and generalizability of the models to other populations and healthcare settings. Second, most original studies exhibited methodological flaws in statistical analysis, such as improper variable handling and insufficient model validation, which may introduce bias and affect the accuracy of performance evaluation. Additionally, although the PROBAST tool is methodologically rigorous, its emphasis on methodological rigor may overshadow the consideration of clinical relevance. Finally, while the literature search covered major databases, the exclusion of grey literature and non-English/Chinese studies restricts the evidence base. Furthermore, the limited number of included studies (*n* = 7) precluded a reliable quantitative assessment of publication bias, potentially limiting the robustness of the conclusions.

## Conclusion

Although existing risk prediction models for PSH show promising discriminative ability, none are currently suitable for routine clinical application due to methodological limitations and the absence of clinical utility assessment. Future models must undergo rigorous prospective validation and incorporate key colorectal cancer-specific factors such as surgical approach, stoma route, neoadjuvant therapy, sarcopenia, prophylactic mesh use, and surgeon experience to enable effective risk-stratified prevention and improve patient outcomes.

## Supplementary Information

Supplementary Material 1

## Data Availability

Not applicable.
