# Supplementary material for: Risk prediction model for parastomal hernia after colorectal cancer surgery: systematic review and meta-analysis
Source: BMC Surg. 2026 Apr 27;26:406. doi: 10.1186/s12893-026-03782-7 (PMC13262515; doi:10.1186/s12893-026-03782-7)
Supplement: Supplementary file 1 — Supplementary Material 1 [file 12893_2026_3782_MOESM1_ESM.docx]

**Supplementary Materials 1**

**Table 1 Retrieval strategies and results in PubMed**

| Search | Query | Items found |
| --- | --- | --- |
| #1 | (((Colostomy[MeSH Terms]) OR (Ileostomy[MeSH Terms])) OR (Ostomy[MeSH Terms])) OR (Enterostomy[MeSH Terms]) | 56682 |
| #2 | (((((((Ostom*[Title/Abstract]) OR (Colostom*[Title/Abstract])) OR (Ileostom*[Title/Abstract])) OR (Loop Ileostom*[Title/Abstract])) OR (Continent Ileostom*[Title/Abstract])) OR (Tube Ileostom*[Title/Abstract])) OR (Incontinent Ileostom*[Title/Abstract])) OR (Enterostom*[Title/Abstract]) | 22220 |
| #3 | #1 OR #2 | 68036 |
| #4 | (hernia[MeSH Terms]) OR (Hernia, Abdominal[MeSH Terms]) | 88160 |
| #5 | (((((Hernia*[Title/Abstract]) OR (Enterocele[Title/Abstract])) OR (Parastomal hernia[Title/Abstract])) OR (Incisional hernia[Title/Abstract])) OR (Abdominal Hernia*[Title/Abstract])) OR (Abdominal Wall Hernia*[Title/Abstract]) | 99360 |
| #6 | #4 OR #5 | 127176 |
| #7 | (((((((nomogram[Title/Abstract]) OR (risk prediction[Title/Abstract])) OR (predict* model[Title/Abstract])) OR (risk model[Title/Abstract])) OR (prediction tool[Title/Abstract])) OR (predictive model[Title/Abstract])) OR (diagnostic model[Title/Abstract])) OR (risk factor*[Title/Abstract]) | 1003830 |
| #8 | #3 AND #6 AND #7 | 162 |

**Table 2 Retrieval strategies and results in Web of Science**

| Search | Query | Items found |
| --- | --- | --- |
| #1 | TS=(Ostom* OR Colostom* OR Ileostom* OR Loop Ileostom* OR Continent Ileostom* OR Tube Ileostom* OR Incontinent Ileostom* OR Enterostom*) | 17669 |
| #2 | TS=(Hernia* OR Enterocele OR Parastomal hernia OR Incisional hernia OR Abdominal Hernia* OR Abdominal Wall Hernia*) | 86025 |
| #3 | TS=(nomogram OR risk prediction OR predict* model OR risk model OR prediction tool OR predictive model OR diagnostic model OR risk factor*) | 5089461 |
| #4 | #1 AND #2 AND #3 | 217 |

**Table 3 Retrieval strategies and results in EMbase**

| Search | Query | Items found |
| --- | --- | --- |
| #1 | ((((ostom* OR colostom* OR ileostom* OR 'loop'/exp OR loop) AND ileostom* OR continent) AND ileostom* OR 'tube'/exp OR tube) AND ileostom* OR incontinent) AND ileostom* OR enterostom* | 28291 |
| #2 | ((((hernia* OR 'enterocele'/exp OR enterocele OR parastomal) AND ('hernia'/exp OR hernia) OR incisional) AND ('hernia'/exp OR hernia) OR abdominal) AND hernia* OR abdominal) AND ('wall'/exp OR wall) AND hernia* | 27507 |
| #3 | ((((((('nomogram'/exp OR nomogram OR 'risk'/exp OR risk) AND ('prediction'/exp OR prediction) OR predict*) AND ('model'/exp OR model) OR 'risk'/exp OR risk) AND ('model'/exp OR model) OR 'prediction'/exp OR prediction) AND ('tool'/exp OR tool) OR predictive) AND ('model'/exp OR model) OR 'diagnostic'/exp OR diagnostic) AND ('model'/exp OR model) OR 'risk'/exp OR risk) AND factor* | 2916914 |
| #4 | #1 AND #2 AND #3 | 62 |

**Table 4 Retrieval strategies and results in Cochrane Library**

| Search | Query | Items found |
| --- | --- | --- |
| #1 | MeSH descriptor: [Colostomy] explode all trees | 258 |
| #2 | MeSH descriptor: [Ileostomy] explode all trees | 316 |
| #3 | MeSH descriptor: [Ostomy] explode all trees | 2182 |
| #4 | MeSH descriptor: [Enterostomy] explode all trees | 651 |
| #5 | (Ostom* OR Colostom* OR Ileostom* OR Loop Ileostom* OR Continent Ileostom* OR Tube Ileostom* OR Incontinent Ileostom* OR Enterostom*):ti,ab,kw | 2159 |
| #6 | #1 OR #2 OR #3 OR #4 OR #5 | 3743 |
| #7 | MeSH descriptor: [Hernia] explode all trees | 4568 |
| #8 | MeSH descriptor: [Hernia, Abdominal] explode all trees | 2460 |
| #9 | (Hernia* OR Enterocele OR Parastomal hernia OR Incisional hernia OR Abdominal Hernia* OR Abdominal Wall Hernia*):ti,ab,kw | 11464 |
| #10 | #7 OR #8 OR #9 | 12035 |
| #11 | (nomogram OR risk prediction OR predict* model OR risk model OR prediction tool OR predictive model OR diagnostic model OR risk factor*):ti,ab,kw | 203062 |
| #12 | #6 AND #10 AND #11 | 34 |

**Table 5 Retrieval strategies and results in EBSCO**

| Search | Query | Items found |
| --- | --- | --- |
| #1 | MH "Colostomy" OR MH "Ileostomy" OR MH "Ostomy" OR MH "Enterostomy" | 31550 |
| #2 | SU (Ostom* OR Colostom* OR Ileostom* OR Loop Ileostom* OR Continent Ileostom* OR Tube Ileostom* OR Incontinent Ileostom* OR Enterostom*) | 444052 |
| #3 | #1 OR #2 | 44057 |
| #4 | MH "Hernia" OR MH "Hernia, Abdominal" | 19411 |
| #5 | SU (Hernia* OR Enterocele OR Parastomal hernia OR Incisional hernia OR Abdominal Hernia* OR Abdominal Wall Hernia*) | 90593 |
| #6 | #4 OR #5 | 90635 |
| #7 | SU (nomogram OR risk prediction OR predict* model OR risk model OR prediction tool OR predictive model OR diagnostic model OR risk factor*) | 2228887 |
| #8 | #3 AND #6 AND #7 | 122 |

**Table 6 Retrieval strategies and results in Zhiwang**

| Search | Query | Items found |
| --- | --- | --- |
| #1 | Risk prediction + prediction model + predictive factors + nomogram + risk score + predictors | 155600 |
| #2 | parastomal hernia | 274 |
| #3 | #1 AND #2 | 9 |

**Table 7 Retrieval strategies and results in Weipu**

| Search | Query | Items found |
| --- | --- | --- |
| #1 | Risk prediction OR prediction model OR predictive factors OR nomogram OR risk score OR predictors | 12786 |
| #2 | parastomal hernia | 435 |
| #3 | #1 AND #2 | 9 |

**Table 8 Retrieval strategies and results in Wanfang**

| Search | Query | Items found |
| --- | --- | --- |
| #1 | Risk prediction OR prediction model OR predictive factors OR nomogram OR risk score OR predictors | 738068 |
| #2 | parastomal hernia | 1679 |
| #3 | #1 AND #2 | 28 |

**Table 9 Retrieval strategies and results in CBM**

| Search | Query | Items found |
| --- | --- | --- |
| #1 | Risk prediction OR prediction model OR predictive factors OR nomogram OR risk score OR predictors | 40051 |
| #2 | parastomal hernia | 635 |
| #3 | #1 AND #2 | 12 |

**Supplementary Materials 2**

**PICOTS Framework**

**Population (P):** Patients who underwent colorectal cancer surgery with creation of an ileostomy or colostomy.

**Index Prediction Model (I):** All prediction models developed or validated for estimating the risk of parastomal hernia in the specified population.

**Comparative Model (C):** Not applicable.

**Outcome (O):** The occurrence of parastomal hernia.

**Timing (T):** The models are intended for use at a defined timepoint following stoma creation (e.g., postoperatively).

**Setting (S):** Hospital environment.

**Supplementary Materials 3**

CHARMS 2014 Relevant items to extract from individual studies in a systematic review of prediction models

| **Domain** | **Key items** | **Reported on page #** |
| --- | --- | --- |
| **SOURCE OF DATA** | Source of data (e.g., cohort, case-control, randomized trial participants, or registry data) | P3 |
| **PARTICIPANTS** | Participant eligibility and recruitment method (e.g., consecutive participants, location, number of centers, setting, inclusion and exclusion criteria) | P3 |
|  | Participant description | P6 |
|  | Details of treatments received, if relevant | Table 1 |
|  | Study dates | P3 |
| **OUTCOME(S) TO BE PREDICTED** | Definition and method for measurement of outcome | P1、3 |
|  | Was the same outcome definition (and method for measurement) used in all patients? | / |
|  | Type of outcome (e.g., single or combined endpoints) | P3 |
|  | Was the outcome assessed without knowledge of the candidate predictors (i.e., blinded)? | / |
|  | Were candidate predictors part of the outcome (e.g., in panel or consensus diagnosis)? | / |
|  | Time of outcome occurrence or summary of duration of follow-up | P6 |
| **CANDIDATE PREDICTORS**  **(OR INDEX TESTS)** | Number and type of predictors (e.g., demographics, patient history, physical examination, additional testing, disease characteristics) | P6、7 |
|  | Definition and method for measurement of candidate predictors | / |
|  | Timing of predictor measurement (e.g., at patient presentation, at diagnosis, at treatment initiation) | / |
|  | Were predictors assessed blinded for outcome, and for each other (if relevant)? | / |
|  | Handling of predictors in the modelling (e.g., continuous, linear, non-linear transformations or categorised) | P6、7 |
| **SAMPLE SIZE** | Number of participants and number of outcomes/events | P6、7 |
|  | Number of outcomes/events in relation to the number of candidate predictors (Events Per Variable) | P6、7 |
| **MISSING DATA** | Number of participants with any missing value (include predictors and outcomes) | P6、7 |
|  | Number of participants with missing data for each predictor | / |
|  | Handling of missing data (e.g., complete-case analysis, imputation, or other methods) | P6、7 |
| **MODEL**  **DEVELOPMENT** | Modelling method (e.g., logistic, survival, neural network, or machine learning techniques) | P6、7 |
|  | Modelling assumptions satisfied | / |
|  | Method for selection of predictors **for inclusion** in multivariable modelling (e.g., all candidate predictors, pre-selection based on unadjusted association with the outcome) | P6、7 |
|  | Method for selection of predictors **during multivariable modelling** (e.g., full model approach, backward or forward selection) and criteria used (e.g., p-value, Akaike Information Criterion) | P6、7 |
|  | Shrinkage of predictor weights or regression coefficients (e.g., no shrinkage, uniform shrinkage, penalized estimation) | / |
| **MODEL**  **PERFORMANCE** | Calibration (calibration plot, calibration slope, Hosmer-Lemeshow test) and Discrimination (C-statistic, D-statistic, log-rank) measures with confidence intervals | P7 |
|  | Classification measures (e.g., sensitivity, specificity, predictive values, net reclassification improvement) and whether a-priori cut points were used | / |
| **MODEL**  **EVALUATION** | Method used for testing model performance: development dataset only (random split of data,  resampling methods e.g. bootstrap or cross-validation, none) or separate external validation (e.g. temporal, geographical, different setting, different investigators) | P7 |
|  | In case of poor validation, whether model was adjusted or updated (e.g., intercept recalibrated, predictor effects adjusted, or new predictors added) | / |
| **RESULTS** | Final and other multivariable models (e.g., basic, extended, simplified) presented, including predictor weights or regression coefficients, intercept, baseline survival, model performance measures (with standard errors or confidence intervals) | P7 |
|  | Any alternative presentation of the final prediction models, e.g., sum score, nomogram, score chart, predictions for specific risk subgroups with performance | P7 |
|  | Comparison of the distribution of predictors (including missing data) for development and validation datasets | / |
| **INTERPRETATION AND DISCUSSION** | Interpretation of presented models (confirmatory, i.e., model useful for practice versus exploratory, i.e., more research needed) | P9-11 |
|  | Comparison with other studies, discussion of generalizability, strengths and limitations. | P9-12 |

**Supplementary Material 4**

**Table 10 PROBAST Assessment Summary for Li et al. (2024)**

| **Author (year)** | **Domain** | | **Signalling Question** | **Response** | **Risk Level** |
| --- | --- | --- | --- | --- | --- |
| Li et al.[18] (2024) | Risk of bias | 1 Participants | 1.1 Data source was a retrospective cohort study | N/PN | - |
|  |  |  | 1.2 Inclusion and exclusion criteria were clearly defined | Y/NY |  |
|  |  | 2 Predictive factor | 2.1 Predictors were defined and assessed consistently | Y/NY | ? |
|  |  |  | 2.2 Not reported | NI |  |
|  |  |  | 2.3 All predictors included in the model were validated | Y/NY |  |
|  |  | 3 Outcome | 3.1 Outcome categorization was appropriate | Y/NY | ? |
|  |  |  | 3.2 Outcome definition was appropriate | Y/NY |  |
|  |  |  | 3.3 Outcome definition excluded the predictors | Y/NY |  |
|  |  |  | 3.4 Outcome definition was consistent for all participants | Y/NY |  |
|  |  |  | 3.5 Not reported | NI |  |
|  |  |  | 3.6 Time interval between predictor assessment and outcome determination was appropriate | Y/NY |  |
|  |  | 4 Analysis | 4.1 Sample size was not justified | N/PN | **-** |
|  |  |  | 4.2 Handling of continuous and categorical predictors was appropriate | Y/NY |  |
|  |  |  | 4.3 All included participants were included in the statistical analysis | Y/NY |  |
|  |  |  | 4.4 Not reported | NI |  |
|  |  |  | 4.5 Predictor selection was based on univariate analysis | N/PN |  |
|  |  |  | 4.6 Not reported | NI |  |
|  |  |  | 4.7 Discrimination and calibration of the prediction model were appropriately assessed | Y/NY |  |
|  |  |  | 4.8 Internal validation methods were correctly applied, and subsequent model performance adjustments were evaluated | Y/NY |  |
|  |  |  | 4.9 Not reported | NI |  |
|  | Applicability | 1 Participants | The study population and clinical setting matched the review question | + | **+** |
|  |  | 2 Predictive factor | The definition, assessment, and timing of predictor measurement matched the review question | + |  |
|  |  | 3 Outcome | The outcome definition, time interval, and analytical approach matched the review question | + |  |
|  | Overall | Risk of bias | **-** | | |
|  |  | Applicability | + | | |

**Notes: Y/NY: Yes/Probably Yes; N/PN: No/Probably No; NI: No Information; ROB: Risk Of Bias; +: Low ROB/Low concern regarding applicability; -: High ROB/High concern regarding applicability; ?: Unclear ROB/Unclear concern regarding applicability.**

**Table 11 PROBAST Assessment Summary for Liu^A^ et al. (2024)**

| **Author (year)** | **Domain** | | **Signalling Question** | **Response** | **Risk Level** |
| --- | --- | --- | --- | --- | --- |
| Liu^A^et al.[17] (2024) | Risk of bias | 1 Participants | 1.1 Data source was a retrospective cohort study | N/PN | - |
|  |  |  | 1.2 Inclusion and exclusion criteria were clearly defined | Y/NY |  |
|  |  | 2 Predictive factor | 2.1 Predictors were defined and assessed consistently | Y/NY | ? |
|  |  |  | 2.2 Not reported | NI |  |
|  |  |  | 2.3 All predictors included in the model were validated | Y/NY |  |
|  |  | 3 Outcome | 3.1 Outcome categorization was appropriate | Y/NY | ? |
|  |  |  | 3.2 Outcome definition was appropriate | Y/NY |  |
|  |  |  | 3.3 Outcome definition excluded the predictors | Y/NY |  |
|  |  |  | 3.4 Outcome definition was consistent for all participants | Y/NY |  |
|  |  |  | 3.5 Not reported | NI |  |
|  |  |  | 3.6 Time interval between predictor assessment and outcome determination was appropriate | Y/NY |  |
|  |  | 4 Analysis | 4.1 Sample size was not justified | N/PN | **-** |
|  |  |  | 4.2 Handling of continuous and categorical predictors was appropriate | Y/NY |  |
|  |  |  | 4.3 All included participants were included in the statistical analysis | Y/NY |  |
|  |  |  | 4.4 Not reported | NI |  |
|  |  |  | 4.5 Predictor selection was based on univariate analysis | N/PN |  |
|  |  |  | 4.6 Not reported | NI |  |
|  |  |  | 4.7 Discrimination and calibration of the prediction model were appropriately assessed | Y/NY |  |
|  |  |  | 4.8 Internal validation methods were correctly applied, and subsequent model performance adjustments were evaluated | Y/NY |  |
|  |  |  | 4.9 The regression coefficients for the predictors were consistent with the reported results. | Y/NY |  |
|  | Applicability | 1 Participants | The study population and clinical setting matched the review question | + | **+** |
|  |  | 2 Predictive factor | The definition, assessment, and timing of predictor measurement matched the review question | + |  |
|  |  | 3 Outcome | The outcome definition, time interval, and analytical approach matched the review question | + |  |
|  | Overall | Risk of bias | **-** | | |
|  |  | Applicability | **+** | | |

**Notes: Y/NY: Yes/Probably Yes; N/PN: No/Probably No; NI: No Information; ROB: Risk Of Bias; +: Low ROB/Low concern regarding applicability; -: High ROB/High concern regarding applicability; ?: Unclear ROB/Unclear concern regarding applicability.**

**Table 12 PROBAST Assessment Summary for Shi et al. (2025)**

| **Author (year)** | **Domain** | | **Signalling Question** | **Response** | **Risk Level** |
| --- | --- | --- | --- | --- | --- |
| Shi et al.[19] (2025) | Risk of bias | 1 Participants | 1.1 Data source was a retrospective cohort study | N/PN | - |
|  |  |  | 1.2 Inclusion and exclusion criteria were clearly defined | Y/NY |  |
|  |  | 2 Predictive factor | 2.1 Predictors were defined and assessed consistently | Y/NY | ? |
|  |  |  | 2.2 Not reported | NI |  |
|  |  |  | 2.3 All predictors included in the model were validated | Y/NY |  |
|  |  | 3 Outcome | 3.1 Outcome categorization was appropriate | Y/NY | ? |
|  |  |  | 3.2 Outcome definition was appropriate | Y/NY |  |
|  |  |  | 3.3 Outcome definition excluded the predictors | Y/NY |  |
|  |  |  | 3.4 Outcome definition was consistent for all participants | Y/NY |  |
|  |  |  | 3.5 Not reported | NI |  |
|  |  |  | 3.6 Time interval between predictor assessment and outcome determination was appropriate | Y/NY |  |
|  |  | 4 Analysis | 4.1 Sample size was not justified | N/PN | **-** |
|  |  |  | 4.2 Handling of continuous and categorical predictors was appropriate | Y/NY |  |
|  |  |  | 4.3 All included participants were included in the statistical analysis | Y/NY |  |
|  |  |  | 4.4 Not reported | NI |  |
|  |  |  | 4.5 Predictor selection was based on univariate analysis | N/PN |  |
|  |  |  | 4.6 Not reported | NI |  |
|  |  |  | 4.7 Discrimination and calibration of the prediction model were appropriately assessed | Y/NY |  |
|  |  |  | 4.8 Internal validation was not performed | N/PN |  |
|  |  |  | 4.9 The regression coefficients for the predictors were consistent with the reported results. | Y/NY |  |
|  | Applicability | 1 Participants | The study population and clinical setting matched the review question | + | **+** |
|  |  | 2 Predictive factor | The definition, assessment, and timing of predictor measurement matched the review question | + |  |
|  |  | 3 Outcome | The outcome definition, time interval, and analytical approach matched the review question | + |  |
|  | Overall | Risk of bias | **-** | | |
|  |  | Applicability | **+** | | |

**Notes: Y/NY: Yes/Probably Yes; N/PN: No/Probably No; NI: No Information; ROB: Risk Of Bias; +: Low ROB/Low concern regarding applicability; -: High ROB/High concern regarding applicability; ?: Unclear ROB/Unclear concern regarding applicability.**

**Table 13 PROBAST Assessment Summary for Dai et al. (2024)**

| **Author (year)** | **Domain** | | **Signalling Question** | **Response** | **Risk Level** |
| --- | --- | --- | --- | --- | --- |
| Dai et al.[20] (2024) | Risk of bias | 1 Participants | 1.1 Data source was a retrospective cohort study | N/PN | - |
|  |  |  | 1.2 Inclusion and exclusion criteria were clearly defined | Y/NY |  |
|  |  | 2 Predictive factor | 2.1 Predictors were defined and assessed consistently | Y/NY | ? |
|  |  |  | 2.2 Not reported | NI |  |
|  |  |  | 2.3 All predictors included in the model were validated | Y/NY |  |
|  |  | 3 Outcome | 3.1 Outcome categorization was appropriate | Y/NY | ? |
|  |  |  | 3.2 Outcome definition was appropriate | Y/NY |  |
|  |  |  | 3.3 Outcome definition excluded the predictors | Y/NY |  |
|  |  |  | 3.4 Outcome definition was consistent for all participants | Y/NY |  |
|  |  |  | 3.5 Not reported | NI |  |
|  |  |  | 3.6 Time interval between predictor assessment and outcome determination was appropriate | Y/NY |  |
|  |  | 4 Analysis | 4.1 Sample size was justified | Y/NY | ? |
|  |  |  | 4.2 Handling of continuous and categorical predictors was appropriate | Y/NY |  |
|  |  |  | 4.3 All included participants were included in the statistical analysis | Y/NY |  |
|  |  |  | 4.4 Missing data were handled using multiple imputation | Y/NY |  |
|  |  |  | 4.5 Predictor selection was not based on univariate analysis | Y/NY |  |
|  |  |  | 4.6 Not reported | NI |  |
|  |  |  | 4.7 Discrimination and calibration of the prediction model were appropriately assessed | Y/NY |  |
|  |  |  | 4.8 Internal validation methods were correctly applied, and subsequent model performance adjustments were evaluated | Y/NY |  |
|  |  |  | 4.9 The regression coefficients for the predictors were consistent with the reported results | Y/NY |  |
|  | Applicability | 1 Participants | The study population and clinical setting matched the review question | + | + |
|  |  | 2 Predictive factor | The definition, assessment, and timing of predictor measurement matched the review question | + |  |
|  |  | 3 Outcome | The outcome definition, time interval, and analytical approach matched the review question | + |  |
|  | Overall | Risk of bias | - | | |
|  |  | Applicability | + | | |

**Notes: Y/NY: Yes/Probably Yes; N/PN: No/Probably No; NI: No Information; ROB: Risk Of Bias; +: Low ROB/Low concern regarding applicability; -: High ROB/High concern regarding applicability; ?: Unclear ROB/Unclear concern regarding applicability.**

**Table 14 PROBAST Assessment Summary for Li et al. (2025)**

| **Author (year)** | **Domain** | | **Signalling Question** | **Response** | **Risk Level** |
| --- | --- | --- | --- | --- | --- |
| Li et al.[22] (2025) | Risk of bias | 1 Participants | 1.1 Data source was a retrospective cohort study | N/PN | - |
|  |  |  | 1.2 Inclusion and exclusion criteria were clearly defined | Y/NY |  |
|  |  | 2 Predictive factor | 2.1 Predictors were defined and assessed consistently | Y/NY | ? |
|  |  |  | 2.2 Not reported | NI |  |
|  |  |  | 2.3 All predictors included in the model were validated | Y/NY |  |
|  |  | 3 Outcome | 3.1 Outcome categorization was appropriate | Y/NY | ? |
|  |  |  | 3.2 Outcome definition was appropriate | Y/NY |  |
|  |  |  | 3.3 Outcome definition excluded the predictors | Y/NY |  |
|  |  |  | 3.4 Outcome definition was consistent for all participants | Y/NY |  |
|  |  |  | 3.5 Not reported | NI |  |
|  |  |  | 3.6 Time interval between predictor assessment and outcome determination was appropriate | Y/NY |  |
|  |  | 4 Analysis | 4.1 Sample size was justified | Y/NY | - |
|  |  |  | 4.2 Continuous variables were converted into two or more categories | N/PN |  |
|  |  |  | 4.3 All included participants were included in the statistical analysis | Y/NY |  |
|  |  |  | 4.4 Not reported | NI |  |
|  |  |  | 4.5 Predictor selection was based on univariate analysis | N/PN |  |
|  |  |  | 4.6 Not reported | NI |  |
|  |  |  | 4.7 Discrimination and calibration of the prediction model were appropriately assessed | Y/NY |  |
|  |  |  | 4.8 Internal validation was not performed | N/PN |  |
|  |  |  | 4.9 The regression coefficients for the predictors were consistent with the reported results | Y/NY |  |
|  | Applicability | 1 Participants | The study population and clinical setting matched the review question | + | + |
|  |  | 2 Predictive factor | The definition, assessment, and timing of predictor measurement matched the review question | + |  |
|  |  | 3 Outcome | The outcome definition, time interval, and analytical approach matched the review question | + |  |
|  | Overall | Risk of bias | - | | |
|  |  | Applicability | + | | |

**Notes: Y/NY: Yes/Probably Yes; N/PN: No/Probably No; NI: No Information; ROB: Risk Of Bias; +: Low ROB/Low concern regarding applicability; -: High ROB/High concern regarding applicability; ?: Unclear ROB/Unclear concern regarding applicability.**

**Table 15 PROBAST Assessment Summary for Liu^B^ et al. (2024)**

| **Author (year)** | **Domain** | | **Signalling Question** | **Response** | **Risk Level** |
| --- | --- | --- | --- | --- | --- |
| Liu^B^et al.[21] (2024) | Risk of bias | 1 Participants | 1.1 Data source was a retrospective cohort study | N/PN | - |
|  |  |  | 1.2 Inclusion and exclusion criteria were clearly defined | Y/NY |  |
|  |  | 2 Predictive factor | 2.1 Predictors were defined and assessed consistently | Y/NY | ? |
|  |  |  | 2.2 Not reported | NI |  |
|  |  |  | 2.3 All predictors included in the model were validated | Y/NY |  |
|  |  | 3 Outcome | 3.1 Outcome categorization was appropriate | Y/NY | ? |
|  |  |  | 3.2 Outcome definition was appropriate | Y/NY |  |
|  |  |  | 3.3 Outcome definition excluded the predictors | Y/NY |  |
|  |  |  | 3.4 Outcome definition was consistent for all participants | Y/NY |  |
|  |  |  | 3.5 Not reported | NI |  |
|  |  |  | 3.6 Time interval between predictor assessment and outcome determination was appropriate | Y/NY |  |
|  |  | 4 Analysis | 4.1 Sample size was not justified | N/PN | - |
|  |  |  | 4.2 Handling of continuous and categorical predictors was appropriate | Y/NY |  |
|  |  |  | 4.3 All included participants were included in the statistical analysis | Y/NY |  |
|  |  |  | 4.4 Not reported | NI |  |
|  |  |  | 4.5 Predictor selection was based on univariate analysis | N/PN |  |
|  |  |  | 4.6 Not reported | NI |  |
|  |  |  | 4.7 Discrimination and calibration of the prediction model were appropriately | Y/NY |  |
|  |  |  | 4.8 Internal validation methods were correctly applied, and subsequent model performance adjustments were evaluated | Y/NY |  |
|  |  |  | 4.9 The regression coefficients for the predictors were consistent with the reported results | Y/NY |  |
|  | Applicability | 1 Participants | The study population and clinical setting matched the review question | + | + |
|  |  | 2 Predictive factor | The definition, assessment, and timing of predictor measurement matched the review question | + |  |
|  |  | 3 Outcome | The outcome definition, time interval, and analytical approach matched the review question | + |  |
|  | Overall | Risk of bias | - | | |
|  |  | Applicability | + | | |

**Notes: Y/NY: Yes/Probably Yes; N/PN: No/Probably No; NI: No Information; ROB: Risk Of Bias; +: Low ROB/Low concern regarding applicability; -: High ROB/High concern regarding applicability; ?: Unclear ROB/Unclear concern regarding applicability.**

**Table 16 PROBAST Assessment Summary for Yang et al. (2025)**

| **Author (year)** | **Domain** | | **Signalling Question** | **Response** | **Risk Level** |
| --- | --- | --- | --- | --- | --- |
| Yang et al.[23] (2025) | Risk of bias | 1 Participants | 1.1 Data source was a retrospective cohort study | N/PN | - |
|  |  |  | 1.2 Inclusion and exclusion criteria were clearly defined | Y/NY |  |
|  |  | 2 Predictive factor | 2.1 Predictors were defined and assessed consistently | Y/NY | ? |
|  |  |  | 2.2 Not reported | NI |  |
|  |  |  | 2.3 All predictors included in the model were validated | Y/NY |  |
|  |  | 3 Outcome | 3.1 Outcome categorization was appropriate | Y/NY | ? |
|  |  |  | 3.2 Outcome definition was appropriate | Y/NY |  |
|  |  |  | 3.3 Outcome definition excluded the predictors | Y/NY |  |
|  |  |  | 3.4 Outcome definition was consistent for all participants | Y/NY |  |
|  |  |  | 3.5 Not reported | NI |  |
|  |  |  | 3.6 Time interval between predictor assessment and outcome determination | Y/NY |  |
|  |  | 4 Analysis | 4.1 Sample size was not justified | N/PN | - |
|  |  |  | 4.2 Handling of continuous and categorical predictors was appropriate | Y/NY |  |
|  |  |  | 4.3 All included participants were included in the statistical analysis | Y/NY |  |
|  |  |  | 4.4 Not reported | NI |  |
|  |  |  | 4.5 Predictor selection was not based on univariate analysis | Y/NY |  |
|  |  |  | 4.6 Not reported | NI |  |
|  |  |  | 4.7 Discrimination and calibration of the prediction model were appropriately assessed | Y/NY |  |
|  |  |  | 4.8 Internal validation methods were correctly applied, and subsequent model performance adjustments were evaluated | Y/NY |  |
|  |  |  | 4.9 The regression coefficients for the predictors were consistent with the reported results | Y/NY |  |
|  | Applicability | 1 Participants | The study population and clinical setting matched the review question | + | + |
|  |  | 2 Predictive factor | The definition, assessment, and timing of predictor measurement matched the review question | + |  |
|  |  | 3 Outcome | The outcome definition, time interval, and analytical approach matched the review question | + |  |
|  | Overall | Risk of bias | - | | |
|  |  | Applicability | + | | |

**Notes: Y/NY: Yes/Probably Yes; N/PN: No/Probably No; NI: No Information; ROB: Risk Of Bias; +: Low ROB/Low concern regarding applicability; -: High ROB/High concern regarding applicability; ?: Unclear ROB/Unclear concern regarding applicability.**

**Supplementary Materials 5**

**
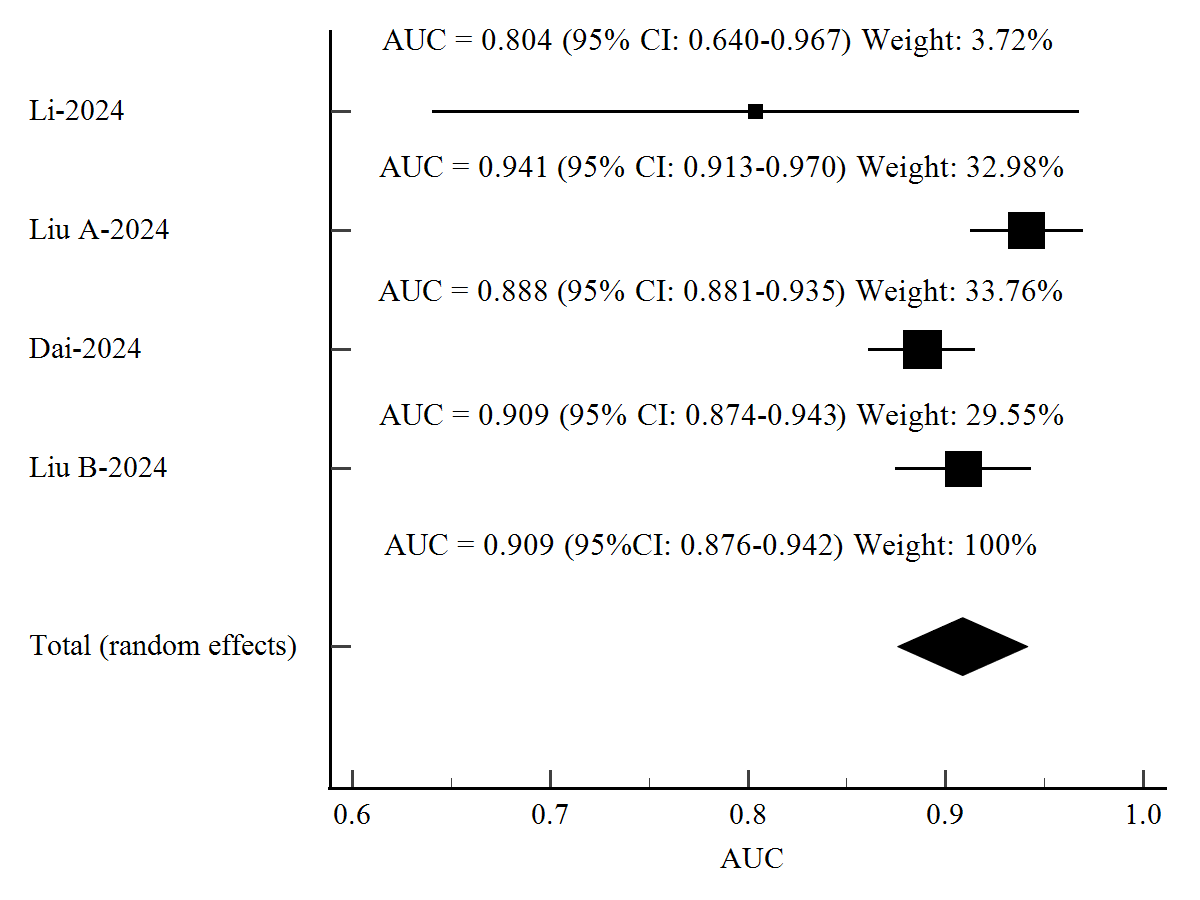
**

**Figure 1 Meta-analysis of AUC in Internal validation groups**


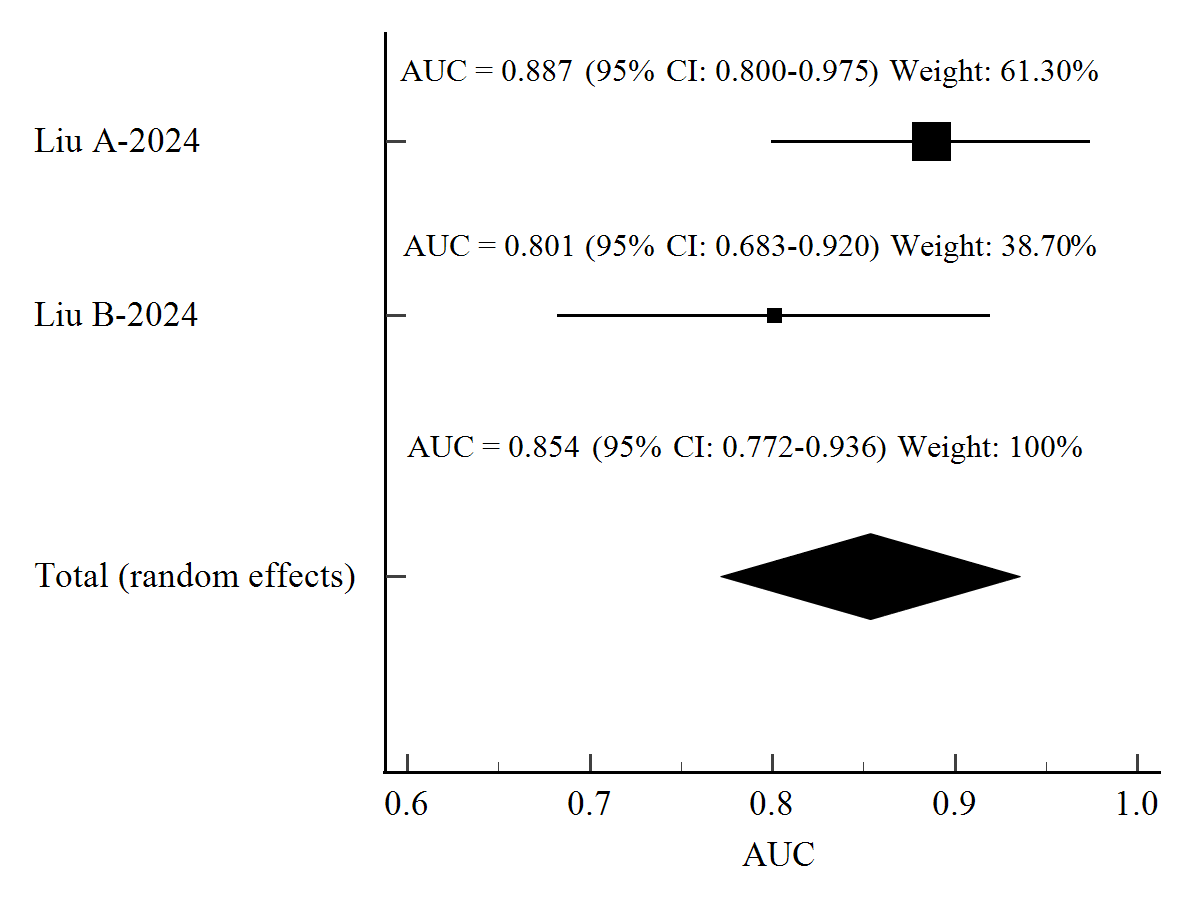


**Figure 2 Meta-analysis of AUC in External validation group**
